# Mini review: Studying epigenomic alterations can shed light on coping and adaptive abilities during heat stress in monogastric livestock

**DOI:** 10.3389/fgene.2025.1561804

**Published:** 2025-08-01

**Authors:** Edoardo Zaccaria, Elianne van der Valk, Soumya K. Kar, Johanna M. J. Rebel, Dirkjan Schokker

**Affiliations:** ^1^ Wageningen Livestock Research, Animal Nutrition, Wageningen, Netherlands; ^2^ Wageningen Livestock Research, Animal Breeding and Genomics, Wageningen, Netherlands; ^3^ Wageningen Bioveterinary Research, Epidemiology, Bioinformatics, Animal Models, and Vaccine Development, Lelystad, Netherlands; ^4^ Adaptation Physiology, Wageningen University, Wageningen, Netherlands

**Keywords:** heat stress, epigenome, livestock, chicken, pig

## Abstract

Epigenomics, a field that studies epigenetic changes on a genome-wide scale, has gained prominence because of its potential to reveal biological mechanisms underlying phenotypes in livestock. Animal production is highly dependent on the interaction between animal genetics, physiology, environment, and management practises. Many of these factors have a bidirectional relationship with the epigenome, as they influence and are influenced by it. This article focuses on the role of epigenetics in the adaptation of livestock to environment, particularly heat stress. Epigenetic changes induced by heat stress have been observed in livestock, resulting in short- and long-term alterations that generally affect production performance and health. Research provides strong evidence that gene expression in livestock is also influenced by epigenetic processes such as DNA methylation, histone modifications, chromatin remodelling, and non-coding RNAs to cope with heat stress. Nutritional interventions are a promising way to mitigate the epigenetic changes induced by heat stress. A better understanding of the molecular mechanisms involved in the regulation of gene expression during heat stress is crucial to identify strategies and interventions that can maintain or even improve the health and productivity of monogastric livestock and adapt their resilience and efficiency to different environmental conditions.

## Introduction

The use of high-throughput technologies in livestock research has increased interest in defining cellular signalling pathways through advances in molecular technology. These include nucleic acid and protein sequencing, as well as metabolite profiling and analysis, with the goal of better understanding the various biological molecules-DNA, RNA, proteins, and metabolites. Often referred to as “omics” studies, this approach provides a better understanding of the mechanisms regulating an animal’s actual physiology and interprets biological mechanisms underlying complex phenotypes, like health and feed efficiency. Enabling omics technology, livestock researchers can estimate breeding values more accurately, thereby assisting the selection of animals (Chakraborty et al., 2022) and finding nutritional strategies or other interventions (Kar, 2017) that enhance animal health and productivity during environmental challenges.

Epigenomics is a field of study that explores the study of epigenetic modifications on a genome-wide scale. From the Aristotelian word epigenesis, the term ‘epigenetics’ was derived, and it was first coined by Conrad Hal Waddington (Waddington, 1942). Epigenetics refers to changes in gene expression or cellular phenotype that occur without altering the underlying DNA sequence. This involves DNA methylation, post-translational modification of histones, but also linked to the regulation of gene expression by non-coding RNAs, genome instabilities or any other force that could modify a phenotype. Enabling the rapid advancements in next-generation sequencing technology, livestock researchers often generate an enormous amount of epigenomic sequencing data that help them to identify and gather epigenomic biomarkers that reveal biological mechanisms underlying complex health phenotypes in livestock. Increasing lines of scientific evidence support the concept that certain acquired traits are derived from environmental exposure during early embryonic and foetal development, i.e., foetal programming, and can even be “memorized” in the germline as epigenetic information and be transmitted to future generations as a non-genetic inherited factors (Zhu et al., 2021; David et al., 2019).

Epigenetic modifications play a crucial role in regulating gene expression and can be influenced by various factors, including developmental processes and environmental stimuli. Heat stress is one of the main environmental factors that negatively affect animal health and welfare, and it will certainly continue to threaten food security (Ross et al., 2015). Specifically, heat stress-induced economic losses result to poor performance, reduced and inconsistent growth, decreased carcass quality, and increased mortality and morbidity (Ross et al., 2015). Despite the fact that all farm animal species are susceptible to heat stress, birds and pigs are particularly sensitive to heat stress due to either lacking or non-functional sweat glands and continues to threaten global sources of animal protein (Ringseis and Eder, 2022; Guo et al., 2018; Nawab et al., 2018; Saeed et al., 2019).

**FIGURE 1:**
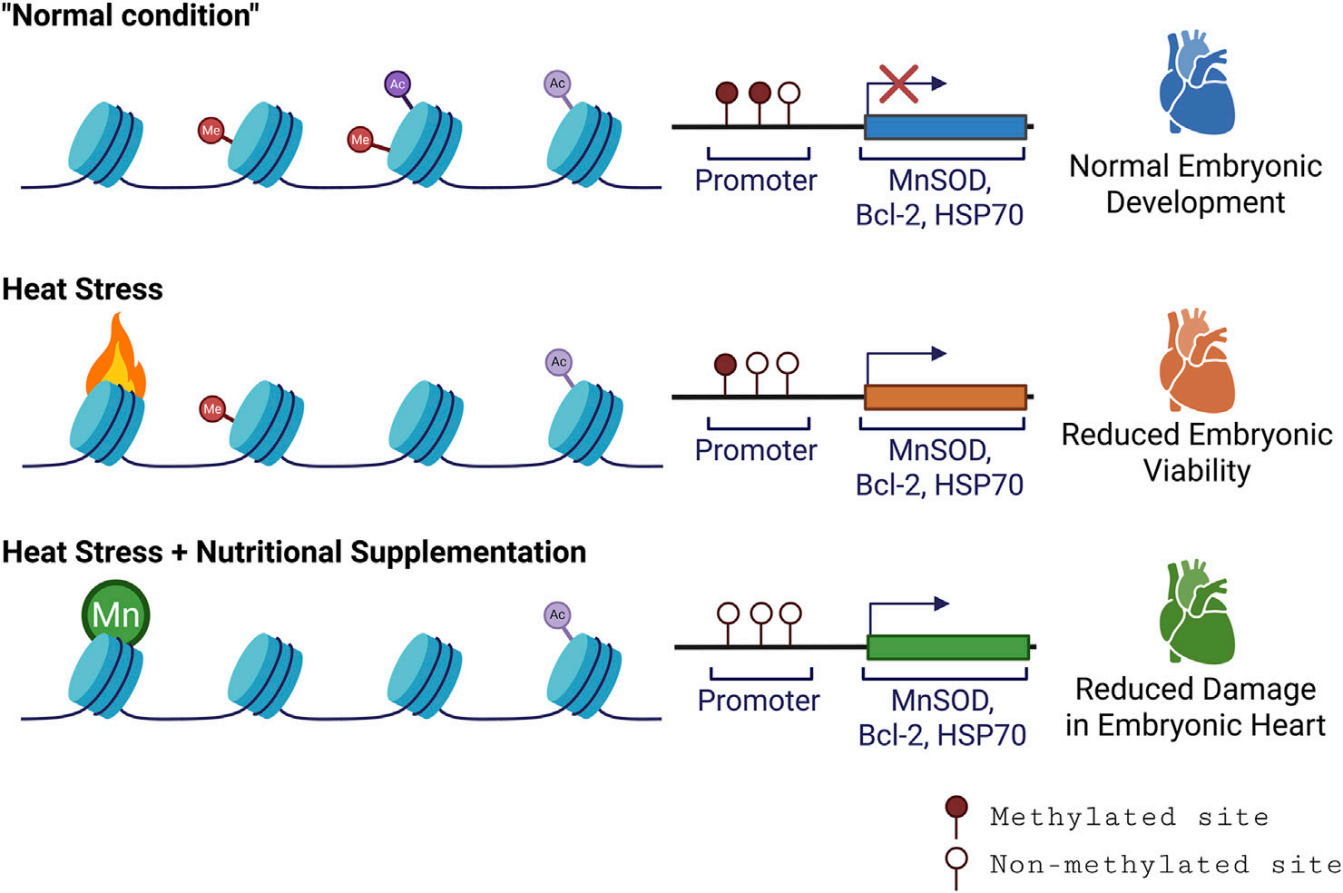
Schematic overview of epigenetic markers, modification, and their effect on host phenotype. Here we highlight that heat stress affects the epigenetic landscape of the embryonic chicken heart. Under the normal condition (baseline methylation), genes involved in antioxidant defence and cell survival (i.e., MnSOD, Bcl-2, HSP70) remain silenced due to DNA methylation and histone modification. Heat stress partially relaxes chromatin structure, but is insufficient alone, leading to reduced embryonic viability. Manganese (Mn) supplementation promotes histone H3K9 hypomethylation and gene activation, leading to improved antioxidant responses and reduced embryonic mortality (Created with BioRender).

Animal production is highly dependent on the interaction between animal genetics, physiology, environment, and management practices, which include housing and feeding, many of which have a bidirectional relationship with the epigenome, as they influence and are influenced by it. Therefore, it is imperative to consider epigenetics as one of the factors responsible for phenotypic variation and resilience in livestock, and it should be considered in livestock breeding, health and disease, management including housing, and provision of nutrition, especially in response to heat stress. While emerging studies indicate that RNA modifications, or RNA epigenetics, also contribute significantly to stress responses in livestock (Xing et al., 2024), only few studies have examined this aspect in monogastrics. In this article, we review the epigenetic coping mechanisms in poultry and pigs (see Figure 1 for a schematic representation). In addition, the impact of changes during embryogenesis and the putative impact of nutrients on animal physiology are discussed.

### Epigenetic processes

By epigenome, we refer to all epigenetic marks on DNA and RNA in a single cell that are generated by epigenetic processes that regulate gene expression and play an important role in genome function and stability. These epigenetic processes are important to the livestock (Pandey et al., 2025), which include DNA and RNA methylation, histone modification, chromatin remodelling, and non-coding RNAs. These processes, including DNA methylation, histone modifications, chromatin remodelling, and non-coding RNAs, play a central role in cellular identity, genome integrity, and environmental adaptation (Sindhu et al., 2024; Ibeagha-Awemu et al., 2022). Epigenetic mechanisms are increasingly recognised as crucial regulators of phenotype in livestock species, impacting traits such as growth, reproduction, immunity, and stress responsiveness (Pandey et al., 2025). In recent years, major technological breakthroughs have transformed our ability to study the animal epigenome with greater resolution and biological relevance. Approaches such as whole-genome bisulfite sequencing (WGBS) for DNA methylation (Yang et al., 2025; Brito et al., 2025), CUT&RUN and CUT&TAG for histone mark profiling (Zhou et al., 2024), and single-cell transcriptomic and chromatin accessibility mapping (e.g., single-cell ATAC-seq, multi-omics platforms) now allow researchers to simultaneously map transcriptional activity, chromatin accessibility, and epigenomic state at the single-cell level in livestock tissues (Li et al., 2024; Tan et al., 2025). These advances have led to unprecedented insights into development, stress responses, and disease in agriculturally important animals. Additionally, the emergence of ideas like epigenetic memory, transgenerational inheritance, and the discovery of functional epitranscriptomic marks (e.g., m6A) in stress responses have shifted the view from static to dynamic and reversible models of gene regulation (Laporta et al., 2024; Henikoff et al., 2025; Sherif et al., 2024; Sajjanar et al., 2025; Xie et al., 2025). With epigenetic memory, we refer to a transient stress that can cause stable changes in gene expression through persistent chromatin marks, such as H3K27ac or DNA methylation, at regulatory regions. It has been demonstrated in chickens conditioned to elevated embryonic incubation temperatures, leading to sustained HSP downregulation and thermotolerance (David et al., 2019; Rosenberg et al., 2022; Halli et al., 2025). Transgenerational epigenetic inheritance, where stress-induced epigenetic marks (e.g., altered methylation in germ cells or early embryos) are passed to offspring, has been observed in poultry and other relevant livestock species (Halli et al., 2025; He et al., 2023). The rise of epitranscriptomics implicates RNA modifications, especially N6-methyladenosine (m6A), as key regulators of mRNA stability, translation, and stress signalling in livestock cells (Chen et al., 2023). A livestock-wide review by Ren et al. (Ren et al., 2024) consolidates evidence that m6A machinery operates across target tissues, liver, muscle, reproductive organs, mediating environmental adaptation and productivity traits.

### DNA methylation

DNA methylation, an addition of a methyl group to a cytosine adjacent to guanine (CpG), is a critical and most studied epigenetic process in mammalian models, catalysed by DNA methyltransferases and demethylated by ten-eleven translocation methylcytosine dioxygenases (TETs) (Unoki, 2019; Wu and Zhang, 2017). Methylation in promoter regions often leads to gene silencing, probably achieved via direct inhibition of transcription factor (TF) binding (Becker et al., 1987; Dantas Machado et al., 2015), while intragenic methylation can enhance transcription and affect splicing (L et al., 2009; Gelfman et al., 2013). This process modulates gene expression in response to environmental stimuli, such as heat stress, across various animal taxa, like worms (*Caenorhabditis elegans)*, fish (*Gasterosteus aculeatus*) and chicken (*Gallus gallus*) (Wan et al., 2021; Metzger and Schulte, 2017; Vinoth et al., 2018). DNA methylation’s role in transcriptional regulation suggests a significant potential contribution to inherited phenotypes, although research predominantly focuses on parent-to-offspring inheritance (Fitz-James and Cavalli, 2022).

### Histone modifications

Histone modification is another epigenetic mechanism with important implications for altering gene expression in response to external stimuli. Histones are a family of proteins (H1-H5) that form structures called nucleosomes via the ordering and packing of the DNA molecule into structural units. Usually, the post-translational modification encompasses methylation, phosphorylation, acetylation, ubiquitylation, or sumoylation of the histone N-terminal tail (Bannister and Kouzarides, 2011; Swygert and Peterson, 2014). While these modifications often upregulate gene expression, the effect is complex, depending on the modification’s nature and location (Jambhekar et al., 2019). Histone modifications, governed by enzymes like histone acetylases and deacetylases, regulate chromatin structure and, thus, transcription factors’ access (Huang et al., 2015; Dose et al., 2011). In heat stress studies, the focus has been on H2B and H3 histones, noting changes in methylation patterns (Marinova et al., 2011; Wu et al., 2020), and multigenerational inheritance of modifications (Klosin et al., 2017).

### Chromatin remodelling (3D genome organisation)

In recent years, more importance has been given to the role of the genome structural organisation and DNA folding in gene regulation, DNA repair, chromosome translocation and cell development (Therizols et al., 2014). Despite DNA folding into nucleosome being well described in the literature (Luger et al., 1997), little is known about how nucleosome interacts with each other or how chromatin folds within the nucleus (Bonev and Cavalli, 2016). Major chromatin remodelling has also been observed upon heat shock, mediated by the Heat Shock Protein 70 (HSP70) and aimed to activate the heat shock response (Khanna et al., 2014). Moreover, local, and quick chromatin changes have been observed within 60 s from temperature elevation, as well as displacement of nucleosomes followed by extensive transcriptional activation of several *HSP* genes (Chowdhary et al., 2019; Kainth et al., 2021). Finally, chromatin remodelling seems to play a pivotal role in animal acclimatisation, increasing resilience to future heat exposures upon moderate heat over long periods (Murray et al., 2022).

### Non-coding RNAs

Non-coding RNAs (ncRNAs) are RNA molecules not translated into proteins but perform various molecular functions. Among the different types of ncRNAs, PIWI-interacting RNA (piRNA), small interfering RNA (siRNA), Long non-coding RNAs (lncRNA), and microRNAs (miRNA) regulate gene expression at the transcriptional and post-transcriptional level. In recent years, a growing interest has been posed on their role in stress response, adaptation, and epigenetic information inheritance (Duempelmann et al., 2020; Czech et al., 2018; O'Brien et al., 2018; Raza et al., 2021; Rossnerova et al., 2020).

### RNA modification

RNA modifications, such as N6-methyladenosine (m6A), represent a dynamic and reversible layer of gene regulation, also referred to as RNA epigenetics. Beyond m6A, more than 160 distinct RNA chemical modifications have been identified, influencing RNA stability, splicing, localization, and translation. Recent breakthroughs reveal that m6A and its associated enzymes modulate the heat stress response in livestock (Cerneckis et al., 2024). For example, maternal heat exposure in pigs alters m6A methylation patterns in neonatal liver and adipose tissue, affecting early fat deposition and metabolic programming (Heng et al., 2019). Similar regulatory roles for m6A have been demonstrated in sheep, where heat stress influences both lipid metabolism and heat shock protein gene expression via m6A-dependent mechanisms (Chen et al., 2023). Although direct evidence in poultry remains limited, cell and a few animal studies indicate that m6A methylation dynamically regulates heat-shock protein pathways in response to acute temperature shifts, suggesting a conserved function across species (Cao et al., 2024). These insights position RNA epigenetics as a promising frontier for enhancing thermotolerance and resilience in monogastric livestock.

## Coping with the changing environment, heat stress and epigenetic modifications

**FIGURE 2:**
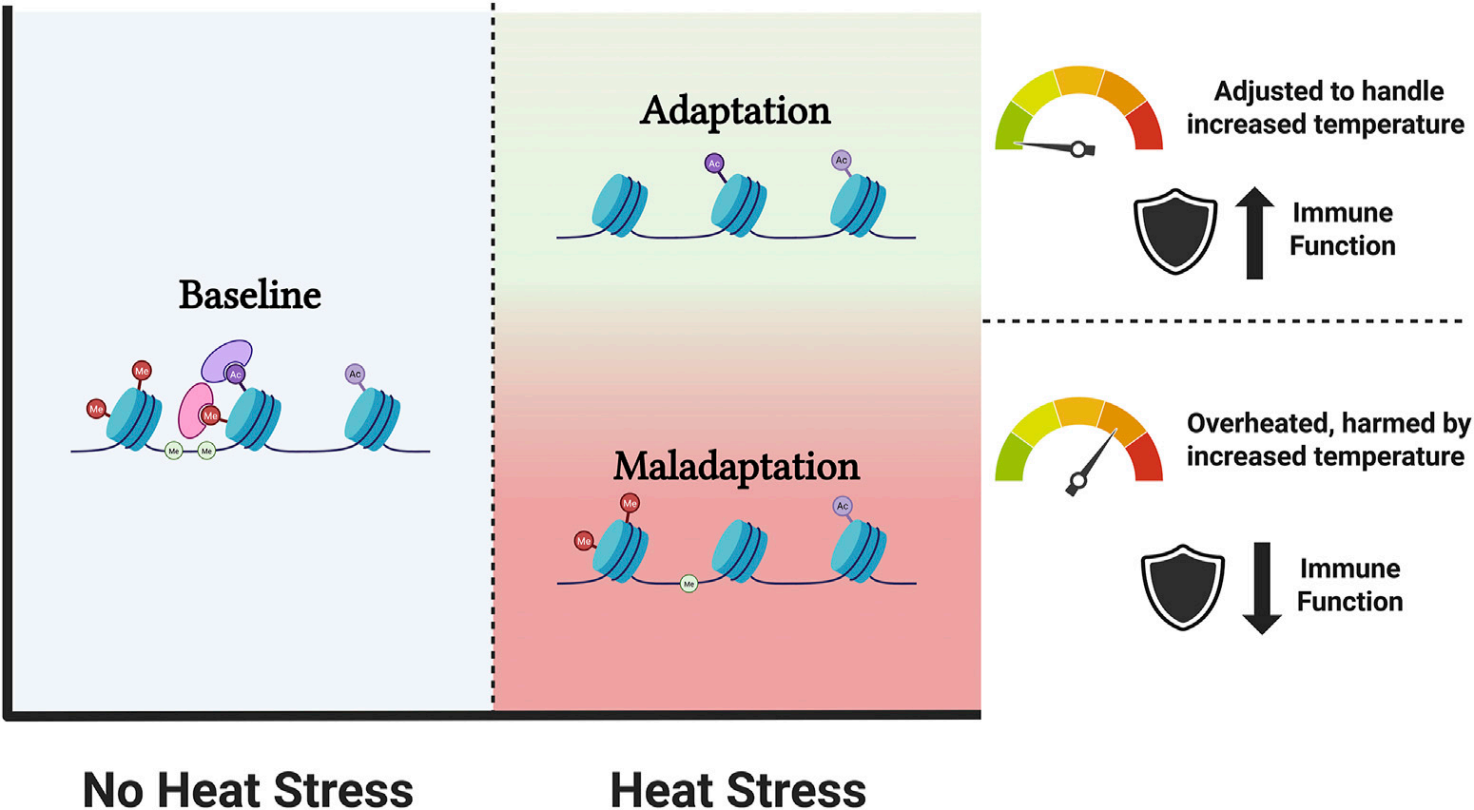
Schematic representation of adaptation to heat stress. The blue zone indicates conditions without heat stress, characterized by a baseline epigenetic landscape. When the animal is experiencing heat stress, adaptation (green zone) or maladaptation (red zone) of the epigenetic landscape occurs. Adapted animal are adjusted to handle increased temperatures often with improved immune function, whereas maladapted animals show overheating, are harmed by the increased temperatures and show a detrimental effect on their immune function. (Created with BioRender).

Heat stress affects livestock productivity, disrupting growth rates, fertility, meat quality, and overall health and welfare. Such stress reprioritizes metabolism, physiology, and behaviour, emphasizing thermoregulation over other activities (Wu et al., 2020; Negrón-Pérez et al., 2019). Over time, heat stress triggers metabolic adaptations and epigenetic modifications that aid in the altered physiological states, from structural and behavioural adjustments to physiological and immunoregulatory changes (Gupta et al., 2022; Goel et al., 2021). Epigenetics plays a major role in this response and eventual adaptation to heat and other stressors, inducing a molecular memory of past experiences (see also Figure 2).

DNA methylation and histone modifications are fundamental regulatory mechanisms found across all biological states, but changes in these epigenetic marks have been observed in animals experiencing heat stress (Wu et al., 2020; Murray et al., 2022; Wang et al., 2019), and can contribute to both short-term adaptation and long-term health consequences. Long and moderate exposure promotes alteration to suboptimal ambient temperature (acclimation to heat or adaptation), leading to easier and faster activation of the heat shock response in the future (Murray et al., 2022). On the other hand, intense heat stress or severe heat injury is associated with long-term health consequences, affecting several metabolic and physiological processes, i.e., immunosuppression, altered heat shock protein (HSP) response, impair cell morphology and apoptosis, which can lead to maladaptation (Wang et al., 2019; Murray et al., 2021; Argaud et al., 2007).

The heat shock response is crucial in cellular and animal survival under stress. This response triggers the rapid increase of HSP that function mainly as molecular chaperones, providing thermotolerance and protection from other stressors (Hietakan et al., 2006). Importantly, HSP expression can be regulated through epigenetic mechanisms, providing an important link between stress adaptation and epigenetics (Tetievsky and Horowitz, 1985; Kisliouk et al., 2017). Temperature-dependent histone-phosphorylation induces the active state of the chromatin that will also be maintained when the stressors are removed, allowing a rapid re-acclimation and promoting a more heat-resilient phenotype (Murray et al., 2022), forming epigenetic memory and affecting HSP expression for several cell cycles (Kisliouk et al., 2017; Dor and Cedar, 2018). For livestock, poultry researchers have shown that by increasing the egg shell temperature during incubation, reduces HSP gene expression post-hatch when encountering a high ambient temperature (Rajkumar et al., 2017).

Dietary strategies and nutritional interventions, including increased dietary fat content and reduced protein intake, have been shown to mitigate heat stress, leading to improved egg production, growth performance, and metabolic efficiency (Poorghasemi et al., 2013; Spencer et al., 2003; Moallem et al., 2010; Kolani et al., 2018). Additives rich in phenolic compounds, flavonoids, and antioxidants also enhance animal welfare during thermal stress (Zimbelman et al., 2010; Oke et al., 2021; Oke, 2018). Moreover, the overall micronutrient composition of the feed must be formulated based on the environmental condition to help the animal to mitigate stress effects. Vitamin C, E, minerals (Na+, K+, and Mg+), and methionine have been reported to produce beneficial effects in goats and chicken (Nwunuji et al., 2014; Saiz del Barrio et al., 2020).

Furthermore, an intriguing concept known as cross-tolerance has been observed in mice and chicken, where early-life heat stress exposure results in increased resilience to other forms of stress (Ben-Nun et al., 2022; Shein et al., 2005). This response is likely due to shared molecular mechanisms across different stress types, leading to a faster and more robust response to subsequent stressors (Ben-Nun et al., 2022; Shein et al., 2005). Rosenberg et al. (Rosenberg et al., 2022; Rosenberg et al., 2020) showed that chicks exposed to high temperatures during embryonic stages displayed transgenerational heat and immunological resilience, emphasizing the potential of epigenetic processes in modulating stress responses (Rosenberg et al., 2022). This transgenerational adaptation, the potential for dietary intervention, and understanding of the heat shock response highlight the importance of multi-disciplinary approaches to tackle the challenges climate change presents to monogastric livestock production.

## Epigenetic impact of heat stress in chicken embryo

During embryogenesis, careful epigenetic remodelling is necessary to avoid developmental defects and ensure healthy development of the embryo. Genomic imprinting involves genes only expressed from the maternal or paternal chromosome in diploid cells and plays crucial roles in early vertebrate development (Barlow, 2011). Heat stress during the early embryonic period can result in lifelong consequences, altering physiological processes, for example, in mouse embryos aberrant methylation imprinting resulted in developmental failure (Zhu et al., 2008). Moreover, these changes may significantly affect embryonic growth, as well as the potential to pass down DNA methylation errors to subsequent generations of livestock (Ross et al., 2017; Ayo et al., 2011; Huber et al., 2020). Previous research has focused primarily on the negative effects of heat stress during embryogenesis (Ross et al., 2017; Ayo et al., 2011; Wettemann and Bazer, 1985). Recent studies have shown that controlled exposure to heat stress during this period can induce epigenetic changes that enhance an animal’s ability to adapt to higher temperatures later in life (Rocha et al., 2022; Piestun et al., 2011).

### Host response to heat stress

Heat shock proteins provide cellular protection, have anti-apoptotic effects, and are synthesized under stress. They play a key role in the heat stress response and adaptation of an animal’ (Parsell et al., 1994). Their expression can be modulated by embryonic heat exposure, and epigenetic processes, such as histone modifications or DNA methylation of HPS promoter regions, may have subsequent effects on heat stress resistance in adulthood (Vinoth et al., 2018; Tetievsky and Horowitz, 1985; Kisliouk et al., 2017).

Epigenomic alteration results shed light on coping and adaptive abilities during heat stress in livestock. Plasticity of the preoptic anterior hypothalamus (POAH) plays an important role in thermoregulation of an animal, and its response may vary depending on heat stress during embryogenesis’ (Tzschentke and Basta, 2002; Griffin et al., 2001). Changes in thermoregulatory activity due to embryonic heat stress may differ between vertebrates. Piglets born from heat-stressed sows had less skeletal muscle and more adipose tissue (Johnson et al., 2015), whereas heat-stressed chickens demonstrated reduced metabolic rates and core temperatures (Piestun et al., 2011; Piestun et al., 2009; Piestun et al., 2008). The plasticity of the POAH in response to environmental stressors has increased the interest in the hypothalamus’s underlying epigenetic changes. Heat stress-induced epigenetic alterations in the hypothalamus may impact the whole body thermoregulation and metabolism. Reduced thyroid gland activity and thyroid hormone T3 levels in heat-stressed chickens emphasize the metabolic shifts, potentially optimizing the animals’ energy efficiency (Vinoth et al., 2018; Piestun et al., 2008; McNabb, 1995).

Results from studies indicate that embryonic heat stress causes epigenetic reprogramming in the hypothalamus, which changes molecular pathways involved in thermoregulation and metabolism. David et al. reported epigenomic modification while investigating the genome-wide distribution of histone modifications in the hypothalamic tissue of heat-stressed chicken embryos. They found modifications in genes related to neurodevelopment, metabolism, and gene regulation that may contribute to environmental stress response (David et al., 2019). These epigenetic changes may contribute to thermal acclimation later in life. Furthermore, the genes like the mammalian ortholog for CREB Binding Protein (*Cbp-1*) and the gene encoding SWI/SNF complex subunit SMARCC2 (*Swsn-1)*, are vital for acetylation and chromatin remodelling, respectively, play central roles in heat stress adaptation (Zhou et al., 2019). In *Caenorhabditis elegans*, inhibiting these genes disrupted the memory of heat adaptation, suggesting their pivotal role in long-term thermoregulatory responses (Zhou et al., 2019; Booth and Brunet, 2016; Benayoun et al., 2015).In addition, embryonic heat stress boosts levels of both corticotropin-releasing hormone (CRH) (Cramer et al., 2019) and brain-derived neurotrophic factor (BDNF) in the hypothalamus (Yossifoff et al., 2008). In fact, extreme heat stress in early postnatal chickens caused an increase in CRH protein levels in the hypothalamus compared to moderate heat stress and control chicken. This increase was associated with hypermethylation in the intron site of the *Crh*, silencing the gene expression (Cramer et al., 2019). Moreover, the expression of the BDNF, an activator of the biochemical pathway involved in heat adaptation (Labunskay and Meiri, 2006; Meiri, 2008) and also controlled by epigenetic processes, in the hypothalamus was increased 3 days after hatching, suggesting a potentially long-lasting effect of embryonic heat stress on several neuronal changes (Yossifoff et al., 2008).

A better understanding of the molecular mechanisms involved in the regulation of gene expression during heat stress is crucial to identify strategies and interventions that can maintain or even improve the health and productivity of livestock and adapt their resilience and efficiency to different environmental conditions.

### Combating heat stress and research opportunities

Heat stress can impact the epigenome during embryonic development, adulthood, and even across generations. As a result, the agricultural sector is searching for ways to lower the impact of the epigenomic changes caused by embryonic heat stress or develop genetic variants of monogastric livestock that are better adapted to heat stress as adult.

Chickens have emerged as pivotal models for understanding the epigenetic repercussions of heat stress (Kisliouk et al., 2010). Exposure to heat, particularly ‘conditioning’ embryos via elevated incubation temperatures, bestows an adaptive epigenetic memory in chickens, thus enhancing their resilience against subsequent heat stress (Zaboli et al., 2017; Yahav and Hurwitz, 1996). This adaptive mechanism speeds up the expression of HSPs and heat shock factor genes and bolsters interleukin and ROS-scavenging protein production (Yahav et al., 2004). Such responses are intricately tied to epigenetic mechanisms, including DNA methylation, histone modifications, and miRNA activity (Ramiah et al., 2022; Kisliouk et al., 2011). Notably, genomic regions associated with chicken domestication display differential methylation patterns, suggesting alterations stemming from selection pressures (Li et al., 2015; Nätt et al., 2012). Conditioning through modified embryonic incubation holds promise as a method to produce poultry that are better equipped to endure heat stress.

In parallel to conditioning embryos with heat, there’s growing interest in “epi-nutrients”. Epi-nutrients are essential nutrients that modulate the epigenome, especially DNA methylation during embryonic development (Kussmann and Bladeren, 2011; Delcurto et al., 2013). These include vital nutrients like vitamin B12, choline, and folate (Mazzio and Soliman, 2014). It is evident that epi-nutrient like folate is present in the bovine oviductal fluid during the oestrous cycle and bovine oviduct epithelial cells express folate receptors and transporters (Garcia et al., 2018). Research has shown that folic acid supplementation in pregnant mice can counter heat stress-induced neural tube defects and alter imprinted gene methylation (Shin and Shiota, 1999). A similar effect has been observed in human embryos when the expecting mothers consumed folic acid (Haggarty et al., 2013). In chickens, manganese-supplemented diets have demonstrated increased hatchability ratios and decreased mortality when exposed to heat stress. This protective effect was tied to heightened expression of the antioxidant enzyme manganese superoxide dismutase, which correlated with global DNA hypomethylation of histone H3 lysine 9 (H3K9) deacetylation in the embryonic heart. The reduced mortality is believed to stem from decreased cell death in the embryonic heart, potentially driven by elevated expression of the anti-apoptotic B-cell lymphoma 2 (Bcl-2) (Zhuo et al., 2017).

While conditioning and epi-nutrients offer encouraging outcomes for countering the epigenetic alterations caused by heat stress, understanding their long-term implications and mechanisms remains an ongoing pursuit. Further studies will help ascertain their full potential in increasing livestock resilience against heat stress, thus aiding in more sustainable agricultural practices.
